# Experiences from the anatomy track in the ontology alignment evaluation initiative

**DOI:** 10.1186/s13326-017-0166-5

**Published:** 2017-12-04

**Authors:** Zlatan Dragisic, Valentina Ivanova, Huanyu Li, Patrick Lambrix

**Affiliations:** Department of Computer and Information Science and Swedish e-Science Research Centre, Linköping University, Linköping, Sweden

**Keywords:** Ontology alignment, Biomedical ontologies, Ontology alignment evaluation initiative

## Abstract

**Background:**

One of the longest running tracks in the Ontology Alignment Evaluation Initiative is the Anatomy track which focuses on aligning two anatomy ontologies. The Anatomy track was started in 2005. In 2005 and 2006 the task in this track was to align the Foundational Model of Anatomy and the OpenGalen Anatomy Model. Since 2007 the ontologies used in the track are the Adult Mouse Anatomy and a part of the NCI Thesaurus. Since 2015 the data in the Anatomy track is also used in the Interactive track of the Ontology Alignment Evaluation Initiative.

**Results:**

In this paper we focus on the Anatomy track in the years 2007–2016 and the Anatomy part of the Interactive track in 2015–2016. We describe the data set and the changes it went through during the years as well as the challenges it poses for ontology alignment systems. Further, we give an overview of all systems that participated in the track and the techniques they have used. We discuss the performance results of the systems and summarize the general trends.

**Conclusions:**

About 50 systems have participated in the Anatomy track. Many different techniques were used. The most popular matching techniques are string-based strategies and structure-based techniques. Many systems also use auxiliary information. The quality of the alignment has increased for the best performing systems since the beginning of the track and more and more systems check the coherence of the proposed alignment and implement a repair strategy. Further, interacting with an oracle is beneficial.

## Background

In recent years many ontologies have been developed and many of those contain overlapping information. Knowledge of the inter-ontology relationships is important in many cases. One example case is when we want to use multiple ontologies, e.g., companies may want to use community standard ontologies and use them together with company-specific ontologies. Other example cases are integration, search and analysis of data in an environment where different data sources in the same domain have been annotated with different but similar ontologies. It has been realized that this is a major issue and much research has been performed on ontology alignment, i.e., finding mappings or correspondences between concepts and relations in different ontologies [42]. The research field of ontology alignment is very active with its own yearly workshop as well as a yearly event, the Ontology Alignment Evaluation Initiative (OAEI, http://oaei.ontologymatching.org/, e.g., [41]), that focuses on evaluating systems that automatically generate correspondence suggestions. Many systems have been built and overviews are found in [87, 99, 123, 144, 145] and at the ontology matching web site http://www.ontologymatching.org. The proceedings of the yearly Ontology Matching workshop contain descriptions of the systems participating in the OAEI as well as summary papers discussing the performance results for these systems in the OAEI.

One of the longest running tracks in the OAEI is the Anatomy track which focuses on two ontologies from the biomedical domain. This domain is one of the earliest adopters of ontologies and a number of large ontologies have been developed and are maintained. This domain manages large volumes of high-complexity data with intricate relationships. Focusing on a particular domain allows the tools to exploit its inherent properties (for instance, it limits the possible meanings of concept labels) and to exploit existing resources as background knowledge. The Anatomy track was started in 2005. In 2005 and 2006 the task in this track was to align the Foundational Model of Anatomy and the OpenGalen Anatomy Model. Since 2007 the ontologies used in the track are the Adult Mouse Anatomy and a part of the NCI Thesaurus. Since 2015 the data in the Anatomy track is also used in the Interactive track of the OAEI.

In this paper we focus on the Anatomy track in the years 2007–2016 and the Anatomy part of the Interactive track in 2015–2016. We describe the data set (ontologies and reference alignment) and the changes it went through during the years as well as the challenges it poses in “OAEI anatomy data and tasks” Section. Further, we give an overview of all systems that participated during these years in the Anatomy track and the techniques they have used (“Participating systems” Section). We discuss the performance results of all systems that participated during these years in the Anatomy track task 1 (“Results in the OAEI anatomy track - task 1” Section), tasks 2 and 3 (“Results in the OAEI anatomy track - task 2 and 3” Section), task 4 (“Results in the OAEI anatomy track - task 4” Section) as well as in the Anatomy part of the Interactive track (“Results in the OAEI interactive track - anatomy” Section). We note that we do not show all the performance results of the individual systems over the years, but instead summarize the general trends. Our paper focuses on the whole period that the track was organized and deals with trends and overviews and multiple systems over the years rather than with individual systems. For results of the individual systems we refer to http://oaei.ontologymatching.org/ as well as the OAEI summary papers^1^ in the proceedings of the Ontology Matching workshops. Further, we summarize our observations^2^ and discuss some possible improvements and changes for the Anatomy track in “Conclusion” Section. We start however with some general information about ontology alignment and the evaluation of ontology alignments.

## Ontology alignment and ontology alignment evaluation

In this section we give some background on ontology alignment. We describe a framework for such systems as well as the measures that are usually used for measuring the performance of ontology alignment systems.

### Ontology alignment

**Fig. 1:**
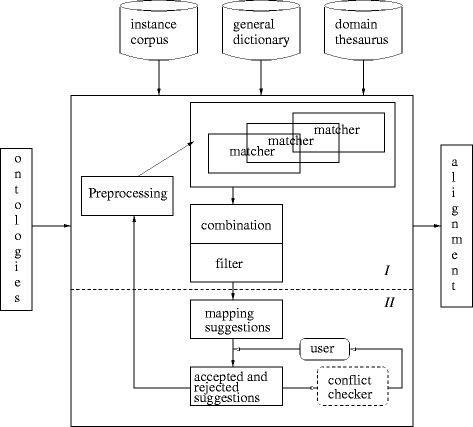
Ontology alignment framework (e.g., [95])

Many ontology alignment systems, although not all, are based on the computation of similarity values between entities in different ontologies and can be described as instantiations of the general framework in Fig. 1. The framework consists of two parts. The first part (I in Fig. 1) computes correspondence suggestions (sometimes called mapping suggestions or candidate mappings). The second part (II) interacts with the user to decide on the final alignment (partly evaluated in the Interactive track). An alignment algorithm receives as input two source ontologies. Part I typically contains different components. A preprocessing component can be used to modify the original ontologies, e.g., to partition the ontologies into mappable parts thereby reducing the search space for finding correspondence suggestions. The algorithm can include several matchers that calculate similarities between the entities from the different source ontologies or mappable parts of the ontologies. They often implement string-based, structure-based, constraint-based and instance-based strategies, as well as strategies that use auxiliary information or a combination of these. Correspondence suggestions are then determined by combining and filtering the results generated by one or more matchers. Common combination strategies are the weighted-sum and the maximum-based strategies. The most common filtering strategy is the (single) threshold filtering. By using different preprocessing, matching, combining and filtering techniques, we obtain different alignment strategies. The result of part I is a set of correspondence suggestions. In part II the suggestions are then presented to the user, a domain expert, who accepts or rejects them. The accepted suggestions are part of the final alignment. In an interactive system the acceptance and rejection of suggestions may also influence further suggestions. Further, in parts I (not in the figure) and II reasoning may be used to check for conflicts and incoherence (see below) and the suggested alignment (and ontologies) may be repaired. There can be several iterations of parts I and II. The output of the alignment algorithm is a set of correspondences between entities from the source ontologies.

### Performance measures

The performance of the systems in the OAEI has typically been evaluated using measures related to the quality of the alignment suggested by the systems (precision, recall and F-measure with respect to a reference alignment) as well as the *run time* of the systems. The *precision* of a system is the ratio of the number of correctly suggested correspondences by the system to the number of suggested correspondences by the system. The *recall* of a system is the ratio of the number of correctly suggested correspondences by the system to the number of correct correspondences according to the reference alignment. *F-measure* is a harmonic mean between precision and recall and is defined as:

$$F_{\alpha} = (1+\alpha) \frac{precision \cdot recall}{\alpha \cdot precision + recall} $$

In addition to these measures the Anatomy track has also computed the recall+ of the systems. As anatomy ontologies often contain similar names, even for different species [64], it is expected that a matcher based on string similarity should do well. Therefore, such a matcher, called StringEquiv, that combines a normalization step and exact string matching, was implemented. The resulting correct suggestions of this matcher were called ’trivial correspondences’ and used as a baseline for recall+. In the most recent reference alignment there are 946 such correspondences out of a total of 1516 correspondences. The *recall+* of a system is the recall of the system on the part of the reference alignment that was not found by StringEquiv and measures thus how well the system finds non-trivial correspondences. According to this definition the recall+ of StringEquiv is equal to 0^3^.

Another measure is the *coherence* of the suggested alignment. An alignment is said to be coherent if the merged ontology containing the original ontologies (in this case AMA and NCI-A) and the alignment is coherent, i.e., does not contain unsatisfiable^4^ concepts.

The data from the Anatomy track is also used in the OAEI Interactive track where a user is simulated using an oracle. In addition to the performance measures above, also the *number of requests to the oracle* is used.

## OAEI anatomy data and tasks

In this section we describe the data sets (ontologies and reference alignment) and their histories as well as the tasks in the Anatomy and Interactive tracks of the OAEI, the particular challenges that this track poses to the alignment systems and the evaluation procedure.

### Ontologies and reference alignment

#### Ontologies

The Adult Mouse Anatomy ontology (AMA) is a part of the Gene Expression Database^5^ and provides a spatial and functional organization of adult mouse anatomical structures^6^. The National Cancer Institute (NCI) Thesaurus^7^ contains more than 100 000 concepts and covers a broad range of topics in cancer research and clinical care. In the OAEI we use a fragment of the NCI Thesaurus containing information about the human anatomy (NCI-A).

In Table 1 we show the evolution of the ontologies used in the Anatomy track. The 2007 version of AMA contained 2744 concepts and 3 object properties. It contained around 4500 subsumption axioms (is-a relations). NCI-A contained 3304 concepts and 2 object properties. There were around 5500 subsumption axioms. The knowledge representation language used for both ontologies was $\mathcal {ALE}$. Both ontologies contained a large number of annotation axioms (AMA - ca 3500, NCI-A - ca 15000). Annotation axioms provide additional information such as provenance information (e.g., creator and owner). In the case of AMA and NCI-A these annotation axioms included properties such as hasSynonym, hasRelatedID and hasDefinition.

**Table 1:** Evolution of AMA and NCI-A and the reference alignment

	AMA	NCI-A	Reference alignment
2007	2744 concepts,	3304 concepts,	1544 equivalence relations
	3 object properties,	2 object properties,	
	ca 4500 subsumption axioms	ca 5500 subsumption axioms	
2008	Same as earlier	Same as earlier	Removed 20 correspondences
2010	Added 12 subsumption axioms	Added 3 subsumption axioms	Weakened 2 correspondences
	Removed 6 subsumption axioms	Removed 3 subsumption axioms	Removed 1 correspondence
		Added 17 disjointness axioms	
2011	Same as earlier	Same as earlier	Added 28 correspondences
			Removed 24 correspondences

The ontologies were changed in 2010. In AMA 12 new subsumption axioms were added and 6 subsumption axioms were removed while in NCI-A 3 subsumption axioms were added and 3 subsumption axioms were deleted. In addition, 17 disjointness axioms were added to the NCI ontology. This required the more expressive knowledge representation language $\mathcal {ALC}$ for NCI-A.

Being developed by different teams and with different purposes in mind AMA and NCI-A exhibit different properties with respect to their structure. Table 2 compares the 2016 versions of the ontologies used in the Anatomy track. The ontologies are comparable in number of concepts but exhibit a large difference in terms of maximum and average depth of leaf concepts. The AMA structure is flatter and approximately a third of the concepts are directly under owl:Thing. NCI-A has a deeper organization and the average depth of concepts for NCI-A is twice as large as for AMA. These two ontologies share a large number of lexically similar labels.

**Table 2:** Comparison between AMA and NCI-A

	AMA	NCI-A
# of concepts	2744	3304
# of direct subconcepts of owl:Thing	1056	7
Maximum depth of the is-a hierarchy	9	13
# equivalent concepts	0	0
# of inner concepts	483	674
# of leaf concepts	2261	2631
Maximum number of direct subconcepts	129	125
# of concepts with one subconcept	74	125
# of concepts with multiple superconcepts	110	277
Average leaves depth		
(= (sum leaf concepts depth)/(# leaf concepts)):	3	6
Average depth (= (sum concepts depth)/(# concepts)):	3	6
Average number of subconcepts (only concepts with subconcepts):	5	5
Average number of subconcepts (all concepts):	1	1

#### Reference alignment

The alignment between AMA and NCI-A was undertaken as part of a project to enable linking data between them. The alignment was developed by using automatic tools as well as a manual approach. As a first step a simple lexical comparison, a preliminary manual comparison by domain experts as well as an approach combining lexical and structural similarity were used [64]. The lexical component in the latter approach uses normalization of terms, exact matching and synonyms from the Unified Medical Language System (UMLS)^8^ Metathesaurus, while the structural component is used as a verification step where only correspondence suggestions which make sense with respect to the structure of the ontologies are retained [6]. The results of the first step were manually validated by domain experts and resulted in 830 correspondences. Further, a number of tools (DAG-OBO-edit [26], Protégé-OWL [124] and COBrA [3]) were selected and used for a further comparative analysis of AMA and NCI-A. It was found that most differences between the ontologies came from design decisions of the hierarchical organization, the coverage of the ontologies and the granularity of the ontologies. Based on this analysis a certain harmonization and extending of the ontologies was performed. This resulted in the versions of the ontologies that were used in the OAEI, and the initial OAEI reference alignment^9^ that contained 1544 equivalence relations (see Table 1).

The reference alignment was modified in 2008 to remove 20 correspondences between concepts which were not part of the ontologies. In 2010, the reference alignment was slightly modified by weakening 2 correspondences (transforming them into subsumption relations) and removing 1 correspondence. The changes were done mostly to produce a coherent alignment as with the pre-2010 versions of the ontologies and the pre-2010 reference alignment the merged ontology containing AMA, NCI-A and the reference alignment was incoherent. The subsumption correspondences were never used in the evaluations. The latest changes in the reference alignment were made in 2011 - 28 correspondences were removed from the reference alignment and 24 new correspondences were added.

In recent years, there have been a number of works, e.g., [5, 64, 71, 97, 98, 102], as well as some personal correspondence^10^ which suggested the existence of missing and wrong is-a relations in the ontologies and missing and wrong correspondences in the reference alignment. However, the evaluation of such mistakes requires domain expertise and so far there has not been such an effort after the latest changes in 2011.

### Tasks

During the years different tasks were introduced in the track:

- Task 1: Align AMA and NCI-A and optimize F-measure.
- Task 2: Align AMA and NCI-A and optimize F-measure with a focus on precision.
- Task 3: Align AMA and NCI-A and optimize F-measure with a focus on recall.
- Task 4: Given a partial reference alignment consisting of all trivial correspondences and 50 non-trivial correspondences, align AMA and NCI-A and optimize F-measure.
- Interactive track: Using an oracle (which may make mistakes), align AMA and NCI-A and optimize F-measure.

In the definition of F-measure, tasks 1, 4 and the interactive track use *α* = 1, while task 2 uses *α* = 5 and task 3 uses *α* = 0.2.

Task 1 has been used in all editions of the OAEI Anatomy track (2007–2016). Tasks 2 and 3 were part of the track during 2007–2010, while task 4 was included in 2008–2010^11^. Since 2011 the *coherence* of the suggested alignment is checked. Tasks 1–4 deal mainly with the non-interactive part of an ontology alignment system (part I in Fig. 1).

Since 2015 the data from the Anatomy track is used in the OAEI Interactive track (run since 2013) which aim is to evaluate the influence of user involvement for interactive alignment tools. It is a first^12^ step towards an evaluation of part II in Fig. 1. In the track users are represented by an oracle and tools can ask the oracle about the correctness of correspondence suggestions and use this information in the generation of other correspondence suggestions.

### Challenges

In the early years the Anatomy track contained the largest ontologies and was therefore the track that evaluated *scalability* of the systems. Nowadays, these ontologies are considered to be medium-sized.

As the two ontologies share a large number of lexically similar labels, string matching-based algorithms do quite well. Therefore, most systems use such algorithms. The challenge is, however, to *combine* these kinds of matchers with other types of matchers to improve the results. Therefore, StringEquiv was used as a baseline matcher to measure the influence of the other types of matchers. Combining matchers in an effective way is not easy and several systems did perform worse than StringEquiv.

As shown in Table 2 the is-a structure of the two ontologies is quite different. One challenge is, therefore, to develop structure-based approaches that can deal with *different is-a structure and granularity*.

The track allows the use of *background information*. Systems need to find appropriate external sources and use them effectively. These external sources may be domain specific or contain general information. The sources may also be incomplete and contain errors.

Task 4 was the only task in any of the OAEI tracks that evaluated the *use of a given partial reference alignment* in the computation of new correspondence suggestions. The partial reference alignment could be used in the preprocessing, computation or filtering components of the systems and new strategies needed to be developed. Task 4 was, however, a difficult task. As the trivial correspondences are given, string-based matching does not give an improvement. Further, given the fact that the partial reference alignment contains only a few non-trivial correspondences, machine learning-based matchers are likely to fail. As the is-a structure of AMA and NCI-A is not complete, structure-based approaches can also not be used to their full potential.

In the Interactive track there are several challenges. The first challenge is to develop strategies for *deciding which correspondence suggestions to show to the oracle*. These questions should be important for the quality of the final alignment. However, there should not be so many questions as to overload the oracle. There should also be not too much *waiting time* between the questions. Then strategies for *using the validation decisions of the oracle* should be developed. This is similar to task 4, but in this case the system has decided which correspondences could be part of the partial reference alignment and additionally, there are also validation decisions about non-correct correspondences. A further challenge in this track is that the systems need to deal with an *oracle that may make mistakes*.

### Evaluation procedure

In the period 2007–2010 the full reference alignment was not publicly available and all tests were done blind. The authors of the tools were provided with the ontologies and were asked to produce an alignment which was then sent to the organizers of the track. The organizers would then evaluate and compare the performance of the tools. In 2010 the SEALS platform^13^ was introduced in the evaluation process for the Anatomy track. SEALS provides an evaluation framework where participants register and upload their tools to the portal. While the reference alignment was still not available, the tools could be run through SEALS and the results for the tool would be directly available. The use of SEALS also meant that the organizers could publish certain tests while keeping other tests blind. In addition to receiving the results directly, the fact that the tools were required to be uploaded made it possible to run all tools on a single hardware which made the comparison of run times possible. Since 2011 the reference alignment has been publicly available.

Initially, the authors of the tools could decide in which track to participate, which made it possible to have specialized tools for certain type of task, e.g., matching biomedical ontologies. However, from 2011 all tools are evaluated in all tracks.

## Participating systems

In this section we give an overview of the participation in the Anatomy track and discuss the techniques used by the different systems.

### Participation

In total ***50 different tools*** (not including different versions of the tools) have been evaluated from 2007 to 2016 in the Anatomy track. The numbers of participants for specific years is given in Table 3. During 2007–2011 around 10 tools participated each year. During 2012–2016 the number of participants has varied from 20 tools in 2013 to 10 tools in 2015.

**Table 3:** Number of participating systems in the OAEI Anatomy track during 2007–2016

Year	Number of distinct tools	Number of tools including different versions
2007	11	11
2008	8	9
2009	10	10
2010	10	10
2011	10	11
2012	14	17
2013	16	20
2014	5	10
2015	11	15
2016	10	13

Tables 4 and 5 show the participants and the years in which they participated. The table lists only the participations in the Anatomy track. During the years that systems were allowed to choose tracks, some systems may have chosen to participate in Anatomy during some years, and not during other years. The latter are not taken up in the table. Further, we only mark a participation in the case of a successful evaluation, i.e, the system returned results within the for that year predefined time frame.

**Table 4:** Participating systems (with different versions) in the OAEI Anatomy track 2007–2016 (part 1)

System	2007	2008	2009	2010	2011	2012	2013	2014	2015	2016
AgreementMaker [14]	[147]		[15]	[13]	[16]					
ALIN [56]										[19]
AML, AML_bk ^2013^ [50, 51]							[49]	[47]	[46]	[48]
Anchor-Flood [143]		[141]	[142]							
AOAS [6, 169]	[168]									
AOT, [NA]										
AOTL								[91]		
AROMA [22]		[23]	[24]		[25]	$\checkmark $				
ASMOV [77]	[74]	[75]	[76]	[78]						
BLOOMS [NA]				[129]						
CIDER-CL [155]							[53]			
CODI [121]				[122]	[69]	$\checkmark $				
COMMAND [113]									$\checkmark $	
CroMatcher [58]									[57]	[59]
CSA [NA]					[154]					
DKP-AOM, [45]										
DKP-AOM-Lite									[43]	[44]
DSSim [114]	[115]	[117]	[116]							
Eff2Match [NA]				[12]						
Falcon-AO[67]	[68]									
FCA_Map[174]										[173]
GeRoMeSuite+SMB [89]				[130]						
GMap [104]									[105]	
GOMMA, [92]										
GOMMA-bk					$\checkmark $	[55]	$\checkmark $			
Hertuda [NA]						[65]	$\checkmark $			
HotMatch [NA]						[21]	$\checkmark $			
IAMA [NA]							[172]			

The references in columns ’2007’ to ’2016’ are to the OAEI papers. When no OAEI paper was published about a system, but it participated we use $\checkmark $. The references in the first column may more fully describe the systems. When not available, we used [NA]

**Table 5:** Participating systems (with different versions) in the OAEI Anatomy track 2007–2016 (part 2)

System	2007	2008	2009	2010	2011	2012	2013	2014	2015	2016
JarvisOM [NA]									✓	
KOSIMap [132]			[131]							
Lily [160, 162]	[158]	[159]	[161]		[156]				[164]	[157]
LogMap, [80, 85]										
LogMapBio ^2014−−2016^,										
LogMapC ^2014−−2015^,										
LogMapLite ^2011−−2016^					[86]	[82]	[83]	[79]	[84]	[81]
LPHOM [110]										[111]
LYAM++ [152]										[153]
MaasMatch [138]					[134]	[135]	[136]	[137]		
MapSSS [NA]					[8]	✓	[11]			
NBJLM [NA]				[163]						
ODGOMS [NA]							[93]			
Optima+ [33, 151]						[150]				
Prior+ [108, 109]	[107]									
RiMOM [103]	[106]	[170]	[171]							
RSDLWB [NA]								[140]	[139]	
SAMBO, [95, 100]										
SAMBOdtf ^2008^	[149]	[101]								
ServOMap, [27]										
ServOMapL ^2012^,										
ServOMBI ^2015^						[4]	[88]		[90]	
SOBOM [NA]			[165]	[166]						
StringsAuto [10]							[11]			
TaxoMap [63]	[167]	[62]	[60]	[61]						
TOAST [148]						[73]				
WeSeE [NA]						[125]	[126]			
WikiMatch [NA]						[66]	✓			
X-SOM [17]	[18]									
XMap, [29]										
XMapGen ^2013^,										
XMapSig ^2013^							[28]	[30]	[31]	[32]
YAM++ [120]						[118]	[119]			

The references in columns ’2007’ to ’2016’ are to the OAEI papers. When no OAEI paper was published about a system, but it participated we use $\checkmark $. The references in the first column may more fully describe the systems. When not available, we used [NA]

Half of the systems has participated more than once. The tools with the most participations (6) are Lily and LogMap. Seven tools have participated 4 times, 6 tools 3 times and 10 tools twice. In the recent instances of the track we can observe an increase in tools which participate with different versions, such as lightweight versions or versions which use background knowledge.

### Alignment techniques

For the overview of the systems in this section we used the papers describing the systems in the OAEI parts of the proceedings of the yearly Ontology Matching workshop. In the case we needed some clarifications we have also looked at the papers referenced in the OAEI papers. For the overview of string-based matchers we also used [10]. We note that some of the participants in the earlier years, may have newer versions of the systems that have features that are not discussed in this paper.

In Table ?? we show the different components of the participating systems. All systems implement part I while some also implement part II and allow iterations. Many systems do some kind of ***preprocessing***. In most of the cases the preprocessing step deals with ***preparing data*** for the matchers. In other cases the systems ***partition the ontologies*** to reduce the search space for the matchers. All systems have a matching component and these are discussed shortly. The ***combination strategies*** are usually ***weighted sum (most common)*** or maximum-based approaches. Some systems use a more advanced approach where the weights for the weighted sum are selected using a neural network (CIDER-CL, X-SOM, XMAP) or a genetic algorithm (XMAPGen), using the overlap between the results of the different matchers (CroMatcher), or using a clustering algorithm (CSA). ***Most filtering*** is performed using a ***single threshold***. SAMBOdtf and X-SOM use a double threshold filtering approach where the correspondences with similarity values between the thresholds are checked with respect to the structure of the ontologies, or are requested to be validated by a user, respectively. Lily uses a maximum entropy approach to calculate a suitable threshold. As the Anatomy track focuses on equivalence correspondences, several systems remove correspondence suggestions when a concept appears in more than one suggestion, for instance, by using a stable marriage algorithm. Early debugging approaches check for such things as criss-cross patterns. However, this does not mean that coherent alignments are generated. Later debugging approaches detect incoherence and also compute repairs. Most debugging appears after the generation of an initial alignment. In contrast, CODI avoids incoherence during the matching steps using a rule-based approach.

**Table 6:** Analysis of the components of the participating systems

Systems	Basic processes					
	Preprocessing^D/R^	Matching	Combination	Filtering	Debugging	User interaction^*^
AgreementMaker	-	✓	✓	✓	-	✓*
ALIN	-	✓	✓	✓	-	✓
AML, AML_bk	D	✓	✓	✓	✓	✓*
Anchor-Flood	D	✓	✓	✓	-	-
AOAS	-	✓	✓	✓	-	-
AOT, AOTL	-	✓	✓	✓	-	-
AROMA	D	✓	✓	✓	-	-
ASMOV	-	✓	✓	✓	✓	✓
BLOOMS	D	✓	✓	✓	-	-
CIDER-CL	D	✓	✓	✓	-	-
CODI	D	✓	✓	✓	✓	-
COMMAND	-	✓	✓	✓	-	-
CroMatcher	D	✓	✓	✓	-	-
CSA	D	✓	✓	✓	-	-
DKP-AOM, DKP-AOM-Lite	D	✓	✓	✓	✓	-
DSSim	R	✓	✓	✓	-	-
Eff2Match	D	✓	✓	✓	-	-
Falcon-AO	R	✓	✓	✓	-	✓*
FCA-Map	D	✓	-	-	✓	-
GeRoMeSuite+SMB	-	✓	✓	✓	✓	✓*
GMap	-	✓	✓	✓	-	-
GOMMA, GOMMAbk	R	✓	✓	✓	✓	✓(*)^1^
Hertuda	D	✓	-	✓	-	✓
HotMatch	D	✓	✓	✓	-	-
IAMA	D	✓	✓	✓	-	-
JarvisOM	D	✓	✓	✓	-	✓
KOSIMap	D	✓	✓	✓	✓	-
Lily	D	✓	✓	✓	✓	✓*
LogMap, LogMapBio,						
LogMapC, LogMapLite	D,R	✓	✓	✓	✓	✓*
LPHOM	D	✓	✓	✓	-	-
LYAM++	D	✓	-	✓	-	-
MaasMatch	D	✓	✓	✓	-	-
MapSSS	-	✓	✓	✓	-	-
NBJLM	-	✓	✓	✓	-	-
ODGOMS	D	✓	✓	✓	-	-
Optima+	-	✓	✓	✓	-	-
Prior+	D	✓	✓	✓	-	-
RiMOM	D	✓	✓	✓	-	-
RSDLWB	D	✓	✓	-	-	✓*
SAMBO, SAMBOdtf	-	✓	✓	✓	✓	✓*
ServOMap(L), ServOMBI	D	✓	✓	✓	✓	✓
SOBOM	-	✓	✓	✓	-	-
StringsAuto	-	✓	✓	✓	-	-
TaxoMap	D,R	✓	✓	✓	-	-
TOAST	-	✓	-	-	-	-
WeSeE	D	✓	-	✓	-	✓
WikiMatch	D	✓	-	✓	-	-
X-SOM	-	✓	✓	✓	✓	-
XMap, XMAPGen, XMAPSig	-	✓	✓	✓	-	✓
YAM++	D	✓	✓	✓	✓	-

^D/R^ D means that the preprocessing is preparing the **d**ata such as collecting and managing/producing (but not just storing) strings from the concept names and descriptions needed for the matchers, and creating hash tables. Also synonyms may be added or an inference engine can be used for enriching the ontology. R means that the search space for the matchers is **r**educed

^*^ The systems with user interaction that are marked with ’*’ have a user interface

^1^ Systems based on GOMMA have a user interface

As different strategies may be differently effective for aligning different kinds of ontologies, AgreementMaker, GeRoMESuite+SMB and RiMoM introduced recommendation strategies^14^ for the settings of the system, such as weights for combination strategies or thresholds for filters.

Tables 7 and 8 provide an overview of the different matching strategies used by the participating systems.^15^ For the string matching strategies we show the string measures that are used. For the structure-based strategies, constraint-based strategies and instance-based strategies we only show the occurrence in the systems. The use of auxiliary information is shown in Table 9.

**Table 7:** Matching Strategies in the participating systems - 1

System	String-based strategies	Structure-based strategies	Constraint-based strategies	Instance-based strategies
AgreementMaker	SubString, Edit-Distance, TF-IDF	✓	✓	✓
ALIN	SimMetrics API^a^, WS4J API^b^	✓	-	-
AML	Jaccard, I-Sub	✓	✓	✓
Anchor-Flood	Jaro-Winkler	✓	-	✓
AOAS	Jaro-Winkler	✓	-	-
AOT, AOTL	Edit-Distance, Block-Distance,			
	SLIM-Winkler, Jaro-Winkler,	-	-	-
	Smith-Winkler, Needleman-Wunsch			
AROMA	Jaro-Winkler	✓	✓	-
ASMOV	Edit-Distance	✓	✓	✓
BLOOMS	Jaccard, Exact Match, Lin,	-	-	-
	Jaro-Winkler			
CIDER-CL	Soft TF-IDF, Jaro-Winkler	✓	-	-
CODI	Edit-Distance, Jaro-Winkler, Cosine,			
	Smith-Waterman, Jaccard,	✓	✓	✓
	Overlap coefficient			
COMMAND	UMBC similarity Model	✓	-	-
CroMatcher	N-Gram, TF-IDF	✓	✓	✓
CSA	Edit-Distance, Wu-Palmer, TF-IDF	✓	-	✓
DKP-AOM, DKP-AOM-Lite	SimMetrics API^a^	✓	✓	-
DSSim	Jaccard, Jaro-Winkler	✓	-	-
Eff2Match	Exact Match, TF-IDF	✓	-	-
Falcon-AO	I-Sub, TF-IDF	✓	-	-
FCA-Map	Exact Match	✓	-	-
GeRoMeSuite+SMB	Edit-Distance, Jaro-Winkler,	✓	-	✓
	I-Sub, Soft TF-IDF,			
	SecondString Library^c^			
GMap	Edit-Distance, TF-IDF	✓	-	-
GOMMA, GOMMA-bk	Exact Match, N-gram	✓	-	✓
Hertuda	Damerau-Levenshtein^d^	-	-	-
HotMatch	Damerau-Levenshtein^d^	✓	✓	✓
IAMA	Edit-Distance	-	-	✓

^a^SimMetrics API is a Java library that includes such string metrics as Jaccard, Jaro-Winkler and N-gram

^b^WS4J (WordNet Similarity for Java) is a Java API containing string metrics like Wu-Palmer, Jiang-Conrath and Lin

^c^SecondString library is a package containing string metrics such as Edit-Distance, Jaro, TF-IDF

^d^Damerau-Levenshtein is a variant of Edit-distance that adds adjacent symbols’ transpositions into the distance measures

**Table 8:** Matching strategies in the participating systems - 2

System	String-based strategies	Structure-based strategies	Constraint-based strategies	Instance-based strategies
JarvisOM	Cosine, WuPalmer, Lin, N-gram	-	-	-
KOSIMap	SimMetrics API^a^, Degree of commonality coefficient	✓	✓	-
Lily	Edit-Distance	✓	✓	✓
LogMap	I-Sub	✓	-	✓
LPHOM	I-Sub, Mongue-Elkan,	-	-	-
	3-Gram, Jaccard, Lin			
LYAM++	SOFT TF-IDF, Jaccard	✓	-	-
MaasMatch	Cosine, Edit-Distance, Jaccard,	✓	-	✓
	3-Gram, Longest Common Substring			
MapSSS	Edit-Distance, Choice based on [10]	✓	✓	-
NBJLM	Set of words-level	✓	-	-
ODGOMS	Longest Common Subsequence, SMOA, TF-IDF	✓	-	-
Optima+	Lin, Smith-Waterman,	✓	-	-
	Needleman-Wunsch			
	Inverse Edit-Distance			
Prior+	Edit-Distance	✓	-	-
RiMOM	Edit-Distance, Cosine	✓	-	✓
RSDLWB	Jaccard, Substring	✓	✓	-
SAMBO, SAMBOdtf	Edit-Distance, 3-Gram	✓	-	✓
ServOMap	Edit-Distance,	✓	-	-
	I-Sub, Q-Gram, TF-IDF,			
	Monge-Elkan, Jaccard			
SOBOM	I-Sub	✓	-	-
StringsAuto	Choice based on [10]	-	-	-
TaxoMap	Lin, 3-gram	✓	✓	-
	Degree of commonality coefficient			
TOAST	✓^b^	✓	-	-
WeSeE	Edit-Distance, TF-IDF	-	-	-
WikiMatch	Jaccard	-	-	-
X-SOM	Edit-Distance, Jaro	✓	-	✓
XMap	Edit distance, Jaro-Winkler,	✓	✓	-
	N-gram, Jaccard, Cosine			
YAM++	Tversky^c^, TF-IDF	✓	-	✓

^a^SimMetrics API is a Java library that include such string metrics as Jaccard, Jaro-Winkler and N-gram

^b^No information found on actual used metrics

^c^Tversky is a similarity metric on string sets

**Table 9:** Use of auxiliary information by the participating systems

System	Background knowledge						
	UMLS	Uberon	BioPortal	MeSH	FMA	WordNet	Other
AgreementMaker	✓	✓	-	-	-	✓	-
ALIN	-	-	-	-	-	✓	-
AML	✓	✓		✓		✓	-
Anchor-Flood	-	-	-	-	-	✓	-
AOAS	✓	-	-	-	✓	-	-
AOT, AOTL	-	-	-	-	-	✓	-
ASMOV	✓	-	-	-	-	✓	-
COMMAND	✓	-	-	-	-	✓	-
CroMatcher	-	✓	-	-	-	✓	-
CSA	-	-	-	-	-	✓	-
DKP-AOM	-	-	-	-	-	✓	-
DSSim	-	-	-	-	-	✓	-
Eff2Match	-	-	-	-	-	✓	-
GOMMA	✓	✓	-	-	✓	-	-
GeRoMeSuite+SMB	-	-	-	-	-	✓	-
Hotmatch	-	-	-	-	-	-	API lanes^a^, WikiPedia,
							Big Huge Thesaurus^b^
JarvisOM	-	-	-	-	-	✓	Apache Lucene^c^
IAMA	-	-	-	-	-	-	Apache Lucene^c^
Lily	-	-	-	-	-	-	Web search (Google)
LogMapBio	-	-	✓	-	-	-	-
LYAM++	-	✓	-	-	-	-	BabelNet^d^
MaasMatch	-	-	-	-	-	✓	-
MapSSS	-	-	-	-	-	-	Google
NBJLM	-	-	-	-	-	✓	-
Optima+	-	-	-	-	-	✓	-
RiMOM	✓	-	-	-	-	✓	Wiki Pages
RSDLWB	-	-	-	-	-	✓	DBpedia^e^
SAMBO	✓	-	-	-	-	✓	-
ServOMap	-	-	-	-	-	✓	Apache Lucene^c^
TaxoMap	-	-	-	-	-	✓	-
TOAST	-	-	-	-	-	✓	-
WeSeE	-	-	-	-	-	-	Microsoft Bing Search
							JFreeWebSearch^f^
WikiMatch	-	-	-	-	-	-	WikiPedia
XMap	✓		-	-	-	✓	-
X-SOM	-	-	-	-	-	✓	Google
YAM++	-	-	-	-	-	-	Apache Lucene^c^

^a^ API lanes is a tool used for natural language processing and text mining

^b^ Big Huge Thesaurus is a dictionary including synonyms

^c^ Apache Lucene used for indexing is a software library for Information Retrieval

^d^ BabelNet is a multilingual encyclopedic dictionary

^e^ DBPedia is a database in which all data is extracted from information from Wikipedia

^f^ JFreeWebSearch is a free library to perform searches on the web

The most commonly used ***matching*** approaches are the string-based approaches. Several ***string similarity metrics*** are frequently used, among which Edit-Distance, TF-IDF or Soft TF-IDF, Jaro-Winkler, NGram or QGram, and Jaccard. We do not discuss the different metrics, but refer for definitions to a larger study from 2013 about the use of these kinds of metrics for ontology alignment [10]. That study suggested that for biomedical ontologies, if we are interested in a high precision then edit distance (Levenshtein) is a good choice. When focusing on high recall or high F-measure, we should consider Jaccard, Soft Jaccard, and Soft TF-IDF. Most of the systems participating after 2013 have used one or more of the recommended matchers.

Regarding ***structure-based strategies***, the most common approach is ***similarity propagation*** where the similarity between concepts influences the similarity between their parents/ancestors and between their children/descendants. Several systems use a variant of the ***similarity flooding*** [112] which is based on the idea that elements are similar when adjacent elements are similar. Other systems take the structure into account in the representation of concepts.

The ***constraint-based approaches*** usually take domain restrictions for relations into account when computing similarity values between concepts.

***Instance-based matching strategies*** use instances when computing similarity values between concepts. When instances are not available other data such as documents containing the concept names are sometimes used as if they are instances. As there are no instances given for AMA and NCI-A, although available in several systems, these strategies ***are rarely used*** in the Anatomy track.

Table 9 shows the use of ***auxiliary information*** by the participating systems. Several systems use sources in the ***biomedical domain*** as auxiliary knowledge. Often these sources collected and integrated biomedical information from other sources. Nine tools use UMLS. UMLS contains entities from many well-known vocabularies, such as ICD-10, MeSH, and SNOMED CT. Five tools use Uberon^16^ as background knowledge. Uberon is an integrated cross-species ontology that covers anatomical structures in animals. BioPortal^17^, a repository with 540 ontologies as well as many alignments, is used by one tool.

Also MeSH^18^, a thesaurus used for indexing articles for PubMed, is used by one tool. Two tools use an intermediate ontology, i.e., the Foundational Model of Anatomy (FMA)^19^.

Regarding the non-biomedical resources most tools use ***WordNet*** ^20^, a large lexical database of English. Further, there are a number of systems which use available search tools or knowledge bases. For instance, Google is used in Lily, MapSSS and X-SOM, and Microsoft Bing search in WeSeE. Hotmatch, RiMOM and WikiMatch make use of Wiki-based background knowledge. Apache Lucene, an information retrieval tool, is used for indexing in JarvisOM, IAMA, ServOMap and YAM++.

## Results in the OAEI anatomy track - task 1

In this section we analyze the results from task 1 in the Anatomy track 2007–2016. Given that the ontologies in the track were changed in 2010 we differentiate between results for the evaluations in 2007–2009 and 2010–2016. We have also reanalyzed the alignments produced by the systems in 2010 w.r.t. the latest reference alignment which was released in 2011. The F-measure is around 1 percentage point higher for all the tools in the reanalyzed 2010 version. In 2011 there were two instances of the track. In the results we only consider the results from the second instance^21^ as that one includes the (modified) tools from the first instance in addition to some new tools.

Based on our analysis we discuss trends of the performance of the systems over the years, by looking at the average or mean performances as well as best performances per year. Although different systems participated during different years, this still gives us an idea of the general direction in which the area is moving. Further, we discuss whether systems participating multiple times improve their performance.

### Quality of the alignment - precision, recall, F-measure, recall+

#### Precision, recall, F-measure

**Fig. 2:**
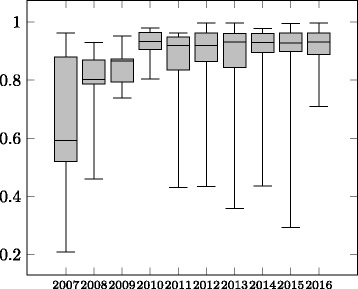
Evolution of precision of the participating systems 2007–2016

**Fig. 3:**
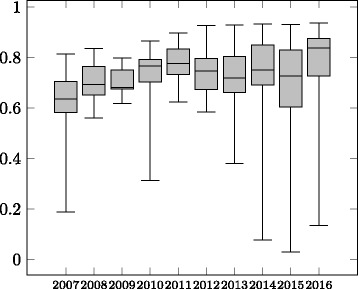
Evolution of recall of the participating systems 2007–2016

**Fig. 4:**
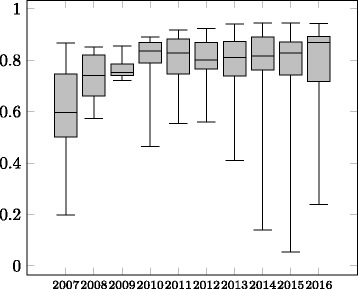
Evolution of F-measure of the participating systems 2007–2016

The evolution of average precision, recall and F-measure is shown in Figs. 2, 3 and 4 in the form of boxplots^22^ for the different years. In the first four years the systems had an almost linear increase in average F-measure over the years. During these years, the improvement was more significant with respect to the average precision. The standard deviation has also decreased in these four years. During 2011–2016, all systems participating in the OAEI were evaluated in all the tracks which caused a decrease in the average F-measure as not all systems were focusing on the Anatomy track, even though the reference alignment was available at that time. In recent years the average precision of the systems was relatively stable while the average recall has experienced a slight drop causing the drop in the average F-measure of the systems.

When considering only the ***best performing tool*** (end of the top whiskers in the boxplots in Figs. 2, 3 and 4) in each year, we observe that until 2012 there has been ***steady increase in performance***. From 2013 the best performing system was AML and its performance in the track has changed very little over the recent years. Similar to the case of average F-measure, ***the increase in F-measure is mainly due to improvements in recall***. The precision results of the best systems in the early days of the track are comparable with the precision results of the best performing systems in recent years.

#### Recall+

**Fig. 5:**
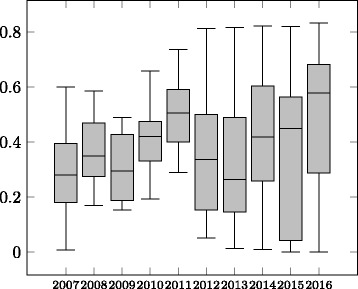
Evolution of recall+ of the participating systems 2007–2016

This measure evaluates the ability of a tool to identify non-trivial correspondences. There has been little improvement over the years (Fig. 5). The largest improvement was between 2009 and 2011. However, as with previous measures, there is a drop in 2012 and then the values until 2016 are relatively stable. In 2016 the average recall+ was at similar levels as in 2011 when the maximum average recall+ was achieved. We also note the large range of recall+ values. Some systems do not manage to find any or just a few non-trivial correspondences, while other systems reach a recall+ value of over 0.8.

When considering only the ***best performing tool*** each year, we can see a ***steady increase*** until 2012 with the exception of 2009. After 2012 GOMMA (in 2012) and AML (2013–2016) obtained recall+ values around 0.8. The highest recall+ is 0.832 achieved by AML in 2016. This corresponds to around 100 non-trivial correspondences which were not found by AML.

#### Use of biomedical knowledge

For the systems that participated with a version using biomedical auxiliary sources and a version not using biomedical auxiliary sources, ***the F-measure for the one with biomedical auxiliary sources was always higher***. This was often because the biomedical auxiliary source allowed the systems to find many more non-trivial correspondences.

#### Multiple participation

We have also evaluated the results to check if the systems which participate often improve their results. For this, we evaluated the performance of the 15 systems which participated at least 3 times in the track. If we only consider the first year and the last year of the participation, all tools except one (ServOMap) show improvements w.r.t. the F-measure. We can see that 7 (Lily, LogMap, AgreementMaker, XMap, CODI, DSSim, GOMMA) systems either improved or achieved the same F-measure as in the their previous participation. There are two systems (AML and MapSSS) which improved or kept the same F-measure in all their participations except the last where the drop of F-measure was less than 0.4 percentage points w.r.t. their best result. Other systems (AROMA, ASMOV, MaasMatch, TaxoMap, ServOMap) have slight fluctuations in their performance over the participating years. This is due to the tweaking of the matching algorithms in some cases to increase recall or in other cases to make the tool perform better in other tracks.

#### Combining systems

An interesting question may be whether we can combine different systems to obtain better results. Table 10 shows aggregated results in different ways. The rows ’year-all’ show the results if we use all systems for a given year. The rows ’year-top 3’ show the results if we use the top 3 systems for a given year. In the row ’Union-best’ we use the best system for each year and in the row ’Union-all’ we aggregate the results for all systems during all years. As expected, when using more systems, the recall and recall+ are always higher and the precision always lower than the recall, recall+ and precision of the best used system. Regarding F-measure, whether there is an improvement or not depends on how much the recall is improved and how much the loss of precision is. In general, the F-measure of the combined systems is lower than the F-measure of the best system of a particular year except when we used the top 3 systems in 2010 and 2011. The ’Union-all’ row shows us that there are still some correspondences which were not found by any system.

**Table 10:** Aggregated results for the period 2010–2016

Case	Size	Precision	F-measure	Recall	Recall+
2010 - all	2103	**0.68**	0.791	0.944	0.852
2011 - all	4735	0.311	0.471	0.971	0.923
2012 - all	4114	0.359	0.525	0.975	0.934
2013 - all	4620	0.32	0.482	0.976	0.937
2014 - all	3271	0.448	0.613	0.968	0.914
2015 - all	2421	0.61	0.75	0.974	0.932
2016 - all	2445	0.611	0.754	**0.985**	**0.96**
2010 - top 3	1621	0.861	0.889	0.92	0.789
2011 - top 3	1590	0.892	0.913	0.935	0.831
2012 - top 3	1618	0.887	0.916	0.947	0.859
2013 - top 3	1645	0.884	0.921	0.96	0.894
2014 - top 3	1718	0.852	0.905	**0.965**	**0.908**
2015 - top 3	1738	0.842	0.899	0.965	0.908
2016 - top 3	1624	**0.895**	0.926	0.959	0.892
Union - best	1735	0.847	0.904	0.969	0.918
Union - all	10756	0.14	0.246	0.995	0.986

Best values for the different measures in the ‘top 3’ and ‘all’ categories are in bold face

#### Rarely found correspondences and most common mistakes

In Table 11 we provide a list of ***rarely found correspondences***. There are 8 correspondences which were not identified by any tool in the period 2010–2016. As expected, the majority of these correspondences ***cannot be identified by string matchers***.

**Table 11:** Correspondences rarely found by systems in the period 2010–2016

Source	Label	Target	Label	
MA_0000793	*mammary gland lobule*	NCI_C12480	*Terminal_Ductal_Lobular_Unit*	0
MA_0000868	*geniculate thalamic group*	NCI_C32673	*Geniculate_Body*	0
MA_0001069	*interpeduncular nucleus*	NCI_C12897	*Oculomotor_Nucleus*	0
MA_0001125	*spinal cord ependymal layer*	NCI_C41624	*Remnants_of_the_Central_Canal_of_the_Spinal_Cord*	0
MA_0001627	*stomach smooth muscle*	NCI_C32657	*Gastric_Muscular_Coat*	0
MA_0001744	*penis foreskin*	NCI_C33049	*Male_Prepuce*	0
MA_0002681	*esophagus muscularis mucosa*	NCI_C32539	*Esophageal_Muscular_Coat*	0
MA_0002682	*esophagus muscle*	NCI_C32540	*Esophageal_Muscularis_Mucosa*	0
MA_0001420	*coccygeal vertebra*	NCI_C12696	*Coccyx*	1
MA_0001098	*optic chiasma*	NCI_C33217	*Optic_Commissure*	1
MA_0002607	*glomerular visceral epithelium*	NCI_C33879	*Visceral_Layer_of_Bowman_s_Capsule*	1
MA_0000449	*peritoneal cavity lining*	NCI_C12770	*Peritoneum*	1
MA_0001697	*urinary bladder smooth muscle*	NCI_C32206	*Bladder_Muscular_Coat*	1
MA_0000545	*male reproductive gland/organ*	NCI_C13017	*Male_Genital_Organ*	1
MA_0002616	*kidney interstitium*	NCI_C33459	*Renal_Interstitial_Tissue*	1
MA_0001900	*gastrointestinal system mesentery*	NCI_C33103	*Mesentery*	1
MA_0001547	*large intestine smooth muscle*	NCI_C32927	*Large_Intestinal_Muscular_Coat*	1
MA_0000332	*ileocaecal junction*	NCI_C13066	*Ileocecal_Valve*	2
MA_0001559	*small intestine smooth muscle*	NCI_C33569	*Small_Intestinal_Muscular_Coat*	2
MA_0001696	*urinary bladder serosa*	NCI_C32208	*Bladder_Serosal_Surface*	2
MA_0000889	*pallidum*	NCI_C12449	*Globus_Pallidus*	2
MA_0002585	*efferent arteriole*	NCI_C33457	*Renal_Efferent_Vessel*	2
MA_0002579	*afferent arteriole*	NCI_C33454	*Renal_Afferent_Vessel*	2
MA_0000183	*telencephalon*	NCI_C12512	*Supratentorial_Brain*	2
MA_0002710	*skin muscle*	NCI_C32419	*Cutaneous_Muscle*	3
MA_0001302	*lens anterior epithelium*	NCI_C32108	*Anterior_Surface_of_the_Lens*	4
MA_0000778	*arrector pili smooth muscle*	NCI_C32534	*Erector_Muscle_of_the_Hair*	4
MA_0001422	*cervical vertebra 2*	NCI_C32174	*Axis_of_the_Vertebra*	4
MA_0000231	*spinal ganglion*	NCI_C12462	*Dorsal_Root_Ganglion*	4
MA_0000065	*capillary*	NCI_C32212	*Blood_Capillary*	4
MA_0002567	*corpora quadrigemina*	NCI_C33443	*Quadrigeminal_Body*	4
MA_0001741	*prostate gland smooth muscle*	NCI_C13100	*Prostatic_Muscular_Tissue*	4
MA_0001030	*trigeminal V sensory nucleus*	NCI_C33402	*Principal_Sensory_Nucleus_of_the_Trigeminal_Nerve*	4
MA_0000814	*brain arachnoid matter*	NCI_C49331	*Cerebral_Arachnoid_Membrane*	5
MA_0000013	*hemolymphoid system*	NCI_C41165	*Hematopoietic_and_Lymphatic_System*	5
MA_0000665	*hindlimb skin*	NCI_C12297	*Skin_of_the_Lower_Limb_and_Hip*	5
MA_0000435	*lower respiratory tract*	NCI_C33012	*Lower_Respiratory_System*	5
MA_0001090	*accessory XI nerve spinal component*	NCI_C12911	*Spinal_Accessory_Nerve*	5
MA_0001790	*right lung hilus*	NCI_C49281	*Hilar_Area_of_the_Right_Lung*	5
MA_0001525	*bowel wall*	NCI_C49478	*Intestinal_Wall_Tissue*	5
MA_0000537	*pelvic girdle muscle*	NCI_C33290	*Pelvic_Floor_Muscle*	5
MA_0001352	*medial cuneiform*	NCI_C32840	*Internal_Cuneiform_Bone_of_the_Foot*	6
MA_0000080	*heart myocardium*	NCI_C12371	*Myocardium*	6
MA_0000617	*forelimb skin*	NCI_C12296	*Skin_of_the_Upper_Limb_and_Shoulder*	6
MA_0002677	*parathyroid gland parenchyma*	NCI_C33270	*Parathyroid_Gland_Tissue*	6
MA_0000763	*spleen central arteriole*	NCI_C33596	*Splenic_Arteriole*	6
MA_0000019	*visceral organ system*	NCI_C28287	*Viscera*	6
MA_0001354	*lateral cuneiform*	NCI_C32554	*External_Cuneiform_Bone_of_the_Foot*	6
MA_0001781	*left lung hilus*	NCI_C49253	*Hilar_Area_of_the_Left_Lung*	7
MA_0000953	*hippocampus CA4*	NCI_C32249	*CA4_Field_of_the_Cornu_Ammonis*	7

The ***most common mistakes*** made by the tools in 2010–2016 are given in Table 12. As expected a large number of these correspondences are due to the fact that labels are relatively similar and thus ***string matchers would classify these with high confidence***.

**Table 12:** Most common mistakes in the period 2010–2016

Source	Label	Target	Label	
MA_0000065	*capillary*	NCI_C12685	*Capillary*	87
MA_0000323	*gastrointestinal system*	NCI_C12378	*Gastrointestinal_System*	82
MA_0001996	*medial femoral circumflex artery*	NCI_C52965	*Medial_Circumflex_Artery*	66
MA_0000003	*organ system*	NCI_C12919	*Organ_System*	65
MA_0002054	*superior gluteal artery*	NCI_C32688	*Gluteal_Artery*	63
MA_0001073	*oculomotor III nucleus*	NCI_C12897	*Oculomotor_Nucleus*	56
MA_0002169	*maxillary vein*	NCI_C32855	*Internal_Maxillary_Vein*	56
MA_0002326	*intercostales internus*	NCI_C32848	*Internal_Intercostal_Muscle*	54
MA_0001591	*taste bud*	NCI_C13147	*Taste_Bud_Cell*	52
MA_0001596	*tongue skeletal muscle*	NCI_C49301	*Tongue_Skeletal_Muscle_Tissue*	51
MA_0002740	*trigeminal V principal sensory nucleus*	NCI_C33402	*Principal_Sensory_Nucleus_of_the_Trigeminal_Nerve*	50
MA_0002070	*ulnar artery palmar branch*	NCI_C33826	*Ulnar_Artery_Branch*	47
MA_0000484	*visceral serous pericardium*	NCI_C13164	*Epicardium*	45
MA_0001006	*cerebellum lobule IX*	NCI_C12232	*Uvula*	45
MA_0001504	*symphysis*	NCI_C32061	*Amphiarthrosis*	45
MA_0002754	*neocortex*	NCI_C12443	*Cortex*	44
MA_0002695	*large intestine wall*	NCI_C32931	*Large_Intestinal_Wall_Tissue*	44
MA_0000998	*cerebellum lobule I*	NCI_C40373	*Lingula*	44
MA_0001176	*intercostal nerve trunk*	NCI_C32825	*Intercostal_Nerve*	41
MA_0002320	*iliocostalis thoracis*	NCI_C32763	*Iliocostal_Muscle*	40
MA_0001036	*dorsal motor nucleus of vagus X nerve*	NCI_C32475	*Dorsal_Nucleus_of_the_Vagus_Nerve*	40
MA_0002474	*mouth*	NCI_C12421	*Oral_Cavity*	37
MA_0001693	*urinary bladder urothelium*	NCI_C13318	*Transitional_Epithelium*	37
MA_0002132	*hepatic portal vein*	NCI_C33343	*Portal_Vein*	36
MA_0002602	*extraglomerular mesangium*	NCI_C32572	*Extraglomerular_Mesangial_Cell*	36
MA_0002151	*right internal spermatic vein*	NCI_C52697	*Right_Spermatic_Vein*	35
MA_0000341	*oral region*	NCI_C12421	*Oral_Cavity*	35
MA_0001720	*cuboidal oviduct epithelium*	NCI_C32415	*Cuboidal_Epithelium*	34
MA_0002150	*left internal spermatic vein*	NCI_C52696	*Left_Spermatic_Vein*	34
MA_0000162	*hair root sheath*	NCI_C32711	*Hair_Root*	33
MA_0001505	*joint of girdle*	NCI_C32890	*Joint_of_the_Pelvic_Girdle*	33
MA_0000288	*olfactory receptor nerve*	NCI_C12633	*Olfactory_Receptor_Neuron*	33
MA_0002677	*parathyroid gland parenchyma*	NCI_C48257	*Parathyroid_Gland_Parenchymal_Cell*	33
MA_0001611	*stomach squamous epithelium*	NCI_C12848	*Squamous_Epithelium*	32
MA_0002058	*sural artery*	NCI_C52734	*External_Sural_Artery*	32
MA_0000812	*brain marginal zone*	NCI_C49767	*Marginal_Zone*	31
MA_0001460	*ovary stratum granulosum*	NCI_C33627	*Stratum_Granulosum*	31
MA_0002033	*pulmonary trunk*	NCI_C12774	*Pulmonary_Artery*	30
MA_0000166	*smooth muscle*	NCI_C12437	*Smooth_Muscle_Tissue*	30
MA_0002225	*superficial cervical vein*	NCI_C33666	*Superficial_Vein*	29
MA_0000259	*auricle*	NCI_C12292	*External_Ear*	29
MA_0001984	*internal thoracic artery*	NCI_C52941	*Internal_Mammary_Artery*	29
MA_0002606	*glomerular mesangium*	NCI_C32685	*Glomerular_Mesangial_Cell*	28
MA_0002749	*spinal cord dorsal column*	NCI_C33588	*Spinal_Cord_Column*	28
MA_0000579	*cranial/facial muscle*	NCI_C13073	*Facial_Muscle*	28
MA_0001245	*corneal stroma*	NCI_C33652	*Substantia_Propria*	28
MA_0002433	*anatomic region*	NCI_C12680	*Body_Region*	28
MA_0002149	*internal spermatic vein*	NCI_C53050	*Spermatic_Vein*	27
MA_0002111	*ductus venosus*	NCI_C32611	*Fissure_of_the_Ductus_Venosus*	27
MA_0001454	*vertebra neural canal*	NCI_C33869	*Vertebral_Canal*	27

For example, *Capillary* in NCI-A is a parent concept which subsumes different types of capillaries. The correct correspondence would be *Blood_Capillary* in NCI-A is equivalent to *capillary* in AMA. A similar issue can be found with *gastrointestinal system* in MA and *Gastrointestinal_System* in NCI-A. In addition to these, common mistakes are those when matchers match concepts which should be related via a part-of relation, e.g., *Taste_Bud_Cell* in NCI-A is a part of *taste bud* in MA, *visceral serous pericardium* in MA is a part of *Epicardium* in NCI-A, and *Extraglomerular_Mesangial_Cell* in NCI-A is a part of *glomerual mesangium* in MA. Similarly, in some cases correspondences are related via an equivalence relation when a subsumption relation is more appropriate, e.g., *superficial servical vein* in MA is a *Superficial_Vein* in NCI-A, and *stomach squamos epithelium* in MA is a *Squamos_Epithelium* in NCI-A. Some of these mistakes might be avoided by combining string matchers with structural matchers which in addition to the label take into account the definition of the concept as well child and parent concepts.

### Influence of defects in the ontologies and reference alignment

A closer analysis of the rarely found correspondences in Table 11 shows that there are ***a number of correspondences which may be erroneous in the reference alignment***. For example, if we consider *Coccygeal_vertebra* in NCI-A and *coccyx* in MA, a more obvious relation would be a part-of relation, as coccygeal vetebrae are only a part of coccyx which is formed from five fused or separate coccygeal vertrebae. Similarly, *Prostatic_Muscular_Tissue* in NCI-A can be seen as a part of prostate gland smooth muscle.

Further, there are correspondences which introduce equivalences in the ontologies which might not be correct. For example, correspondences *esophagus muscularis mucosa* ≡*Esophageal_Muscular_Coat*, *esophagus muscle* ≡*Esophageal_Muscularis_Mucosa* and *esophagus muscularis mucosa* ≡*Esophageal_Muscularis_Mucosa* from the reference alignment will make *Esophageal_Muscularis_Mucosa* equivalent to *Esophageal_Muscular_-Coat* in NCI-A and *esophagus muscle* equivalent to *esophagus muscularis mucosa* in AMA. Similarly, correspondence *pallidum* ≡*Globus_pallidus* together with the correspondence *globus pallidus* ≡*Globus_pallidus* from the reference alignment would imply that *globus pallidum* is equivalent to *pallidum* in AMA while they are currently related via a part of relation. Another example is the correspondence between *heart myocardium* and *Myocardium* which together with the correspondence between *myocardium* and *Myocardium* from the reference alignment would make *heart myocardium* and *myocardium* (its superconcept) equivalent in AMA.

In some cases there are correspondences whose concepts have a different cross-reference in the Uberon ontology. For example, for the correspondence *penis foreskin* and *Male_Prepuce* according to the Uberon ontology *prepuce* in AMA should be equivalent to *Male_Prepuce*. NCI-A does differentiate between male and female prepuce but prepuce in AMA is defined as a part of male reproductive system and as such is a better candidate for the correspondence. This also implies that the correspondence between *prepuce* in AMA and *Prepuce* in NCI-A from the reference alignment is incorrect as *Prepuce* in NCI-A is a superconcept of *male prepuce* and *female prepuce*. Another example is the correspondence between *interpeduncular nucleus* and *Oculomotor_Nucleus*. According to the Uberon ontology, the correspondence between *oculomotor III nucleus* and *Oculomotor_Nucleus* is more appropriate.

There are also a number of missing correspondences in the reference alignment. For example, *intercostales internus* should be equivalent to *Internal_Intercostal_Muscle*. An argument for this is also that the correspondence between the parents of these concepts is a part of the reference alignment as well as the correspondence between *intercostales externus* and *External_Intercostal_Muscle*. Similarly, *internal thoracic artery* is a synonym of *Internal_Mammary_Artery* and as such should be part of the reference alignment. The concepts in this correspondence reference the same concept in the Uberon ontology which can be an argument for inclusion to the reference alignment. We have also conducted an analysis of other cross-references in Uberon and have identified that there are in total 62 correspondences whose concepts cross-reference a concept in Uberon and which are not in the reference alignment. However, domain knowledge is needed to identify if these are actually missing in the reference alignment or are mistakes in the Uberon ontology. In Table 12 correspondences which have cross-reference in Uberon are relations between: *oculomotor III nucleus* and *Oculomotor_Nucleus*, *maxillary vein* and *Internal_Maxillary_Vein*, *trigeminal V principal sensory nucleus* and *Principal_Sensory_Nucleus_of_the_Trigeminal_Nerve*, *dorsal motor nucleus of vagus X nerve* and *Dorsal_Nucleus_of_the_Vagus_Nerve* and finally *internal thoracic artery* and *Internal_Mammary_Artery*.

### Quality of the alignment - coherence

**Fig. 6:**
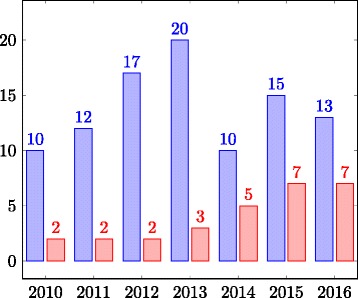
Number of the participating systems that produce a coherent alignment (red bar) w.r.t. to the total number of participants (blue bar)

The changes in the reference alignment and ontologies in 2010 and 2011 which made the merged ontology coherent, made it possible to test the ***coherence*** of the produced alignments. The coherence of generated alignments (Fig. 6) was evaluated for the first time in 2011 when only 3 tools produced a coherent alignment. In 2012, 2 systems out of 17 produced a coherent alignment and in 2013 only 3 out of 20. In the period 2014–2016 ***around half of the systems produced a coherent alignment***.

### Run times

The run times have been evaluated in all years except in 2010. In the first few years of the track run times were reported by the participants directly which meant that the run times were not fully comparable because of the differences in the hardware. In 2013–2016 the same hardware was used so the run times are directly comparable.

**Fig. 7:**
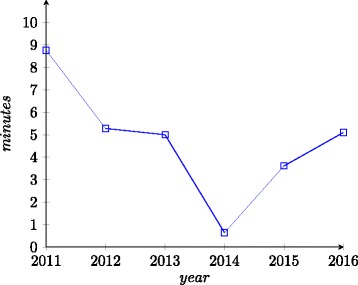
Evolution of run-times (medians) in the period 2011–2016

Before 2011, when systems were tested on only preferred tracks, we can observe significant improvements in run times (Fig. 7). In the first instance of the track the median time was around 4.5 h, where the longest run time was 4 days (Lily). The fastest system in 2007 was Falcon-OA with 12 m. In 2008 the median run time was around 25 m with 17 h for the slowest system (SAMBO) and 1 m for the fastest system (Anchor-Flood). The fastest system in 2009 was AROMA with a run time of 1 m and the slowest was Lily with 99 m. The median run time was around 11 m.

From 2011 and on the trend in run times is not as obvious. In 2011 the median run time was around 9 m. Again there were a number of systems with extremes such as MaasMatch with around 7 h. The following three years the median run times have continued decreasing with medians of 5, 3.5 and 0.6 m, respectively. However, in the last two years, 2015 and 2016, there has been an increase in median run times. In 2015 the median run time was 3.61 m while in 2016 the median was 5.1 m.

### Quality of the alignment versus run times

We have analyzed the performance results and ***do not observe any correlation between the run times of the tools and the quality of the alignments*** they produce.

## Results in the OAEI anatomy track - task 2 and 3

During the four years (2007–2010) that tasks 2 and 3 were organized, in general, the systems could be optimized with a focus on precision and recall. In all cases an improvement in precision led to a drop in recall, and vice versa.

Most systems use different thresholds for the filtering step in the alignment to optimize with a focus on precision (higher threshold) or recall (lower threshold), respectively. Some systems use additional heuristics. e.g., a more flexible matching approach to increase recall, or a more strict approach to increase precision.

## Results in the OAEI anatomy track - task 4

In this task a partial reference alignment consisting of all trivial correspondences and circa 50 non-trivial correspondences, is given and AMA and NCI-A should be aligned focusing on optimizing the F-measure. The aim is to compare different approaches that can take given correspondences into account and evaluate whether they can improve the quality of the alignments using this information.

During the three years this track was organized eight^23^ systems participated: ASMOV (2008–2010), RiMOM (2008), SAMBO (2008), SAMBOdtf (2008), Anchor-Flood (2009), AgreementMaker (2009–2010), TaxoMap (2009) and CODI (2010).

All participants except CODI managed an improvement in precision (up to circa 3 percentage points), while CODI had a very small decrease. This is natural as ***most systems used the partial reference alignment to remove incorrect correspondences***. Only SAMBO, SAMBOdtf, Anchor-Flood and CODI showed an increase in recall. As all non-trivial correspondences are given in the partial reference alignment, an increase in recall means that new non-trivial correspondences were found. The increase or decrease in F-measure is small for all systems.

As the track organizers in 2008–2010 observed^24^ and as we have noted in the “Challenges” section, task 4 is actually hard. The non-trivial correspondences are easily found by string matching algorithms. As the partial reference alignment contains those but only a few non-trivial correspondences, machine learning-based matchers are likely to fail. Further, as shown in [96], the is-a structure of AMA and NCI-A is not complete and thus structure-based approaches can also not be used to their full potential. It is thus not easy to improve the results given the used partial reference alignment. Although the task was run for only three years, it has inspired other work. For instance, a deeper study on the use of partial alignments in ontology alignment inspired by task 4 is found in [96]. The task has also inspired work on completion and debugging of ontologies, e.g., [97].

## Results in the OAEI interactive track - anatomy

In the Interactive track user interactions are simulated using an oracle in the SEALS client. An interactive matching system can present one or a collection of correspondences to the oracle, which will tell the system whether the correspondences are correct or wrong. To simulate the possibility of user errors, the oracle can be set to reply with a given error probability (randomly, from a uniform distribution). Systems were evaluated with four different error rates: 0.0 (perfect oracle), 0.1, 0.2, and 0.3.

In the two years that the Anatomy data set was used six systems participated: AML (2015–2016), LogMap (2015–2016), JarvisOM (2015), ServOMBI (2015), ALIN (2016) and XMap (2016). Not all of these systems have user interfaces, but they all implement an interface to communicate with the oracle.

The different systems use different strategies for using the oracle. AML, LogMap and XMap request feedback on selected mapping suggestions and filter correspondence suggestions based on oracle validations. LogMap interacts with the oracle to decide on correspondence suggestions which are not clear-cut cases. AML employs a query limit and other strategies to minimize interactions with the oracle. XMap asks mainly information regarding true negatives. ServOMBI asks the oracle to validate all of its correspondence suggestions and uses the validations and a stable marriage algorithm to decide on the final alignment. Also ALIN asks the oracle to validate its correspondence suggestions and uses the validations to remove suggestions in conflict with suggestions validated to be correct. Further, new suggestions may be added related to relationships in correct suggestions. JarvisOM is based on an active learning strategy known as query-by-committee. At every iteration JarvisOM asks the oracle for pairs of entities that have the highest disagreement between committee members and lower average Euclidean distance, and at the last iteration, the classifiers committee is used to generate the alignment.

The different strategies influence the number of requests to the oracle. As ServOMBI requests all suggested correspondences to be validated it always requested 1130 interactions. ALIN requested up to 800 interactions. LogMap checked difficult cases and had up to 650 interactions. AML has strategies for reducing interactions and requested 300 interactions. XMap and JarvisOM have very specific strategies. XMap had always 35 interactions, while JarvisOM up to 10.

The ***F-measure of the systems always improves when interacting with a perfect oracle*** compared to no interaction. For most systems there is a raise in precision with similar or slightly lower recall, but ALIN and JarvisOM obtained a large raise in recall.

Although the systems’ F-measures become lower when the oracle is making mistakes, there are still benefits from the interaction. For several of the systems the ***F-measure is still higher when interacting with an oracle that makes mistakes*** than when not interacting at all. Another benefit is, for instance, for ALIN which detects only trivial correspondences in the non-interactive version while using interaction it also detected some non-trivial correspondences. As expected, systems that rely more heavily on interaction are also more influenced by the quality of the oracle.

Regarding the number of unsatisfiable concepts resulting from the alignments we observe some expected variations as the error increases. We note that, with interaction, the alignments produced by the systems are typically larger than without interaction, which makes the repair process harder. The introduction of oracle errors complicates the process further.

Two models for system response times, Shneiderman and Seow, are frequently used in the literature [20]. The request intervals for AML, LogMap and XMap stay under 3 ms for all data sets. ALIN’s request intervals are around 160 ms. ServOMBI often has a request interval around 10 ms, but also has intervals of circa 1 s. JarvisOM’s request intervals become larger for error rates 0.2 and 0.3 with about half of the requests taking above 1.5 s. These ***response times are acceptable*** with respect to the models for system response times.

## Conclusion

We summarize the lessons learned and give possible changes for the future instantiations of the track.

### Summary of observations

On average 10 to 15 systems participated in the Anatomy track with 2013 the top year regarding participation. About half of the tools have participated more than once. Of the 15 systems that participated at least three times almost all systems improved their performance over the years in terms of quality of the alignment.

Many systems implement a preprocessing step. In most systems this step deals with data preparation, while in some systems the step (also) deals with the reduction of the search space for the matchers. The latter is a way for dealing with the *scalability challenge*. The most common combination approach is using weighted sum and the most common filtering approach is using a threshold.

Most systems implement multiple matching strategies and deal with the *combination challenge*. All systems use string-based strategies, although not always the recommendations from [10]. Most systems implement structure-based strategies, often a form of similarity propagation or similarity flooding. Some systems implement constraint-based strategies often based on the domains of relations. Instance-based approaches are not often used in the Anatomy track. About half of the systems deal with the *background information challenge*. The most often used resource is WordNet. Regarding the use of biomedical background knowledge, UMLS is the most used resource. Uberon, BioPortal, MeSH and FMA are used sometimes, mostly by systems focusing on the biomedical tracks in the OAEI. The biomedical auxiliary sources allowed the systems to find more non-trivial correspondences.

More and more systems check the coherence of the proposed alignment and implement a repair strategy.

The quality of the alignment has increased for the best performing systems since the beginning of the track. The improvements in F-measure are usually due to an improvement in recall. The best early systems had a level of precision similar to the best newer systems.

Many of the most commonly found correspondences as well as many of the most common mistakes are correspondences that are easily found by string matchers. Some of the correspondences in the reference alignment may, however, be wrong.

There is a wide spread in run times for the systems. Some systems do not *scale* well. We did not observe any correlation between the run times of the systems and the quality of the alignments they generate.

Given a *partial reference alignment* most systems used it to remove incorrect correspondences. The task that dealt with this challenge ran only for 3 years. A variant of this challenge is now included in the Interactive track.

The Interactive track has not run so often yet and only a few systems participated in the track. Therefore, the solutions to the *user interaction challenges* are diverse. In the current solutions interacting with a perfect oracle always improves the quality of the proposed alignment. Even when the oracle makes mistakes, there are still benefits from the interaction. The waiting times between questions to the oracle are acceptable and according to accepted interaction models.

### Possible future directions

One possible change is to update the ontologies and the reference alignment. There are newer versions of the ontologies and the alignments which could be used (e.g., [64]). Further, we know about mistakes in the ontologies and the reference alignment which should be repaired. For this we will need the help of domain experts, preferably the maintainers of the ontologies. A disadvantage is, however, that we cannot compare the results of the systems historically.

A further possible direction is to evaluate how systems deal with defects. Given ontologies and a reference alignment with defects, systems need to detect and repair these defects. Many systems that do repairing focus on obtaining a coherent alignment, and for this purpose may actually remove correct correspondences [128]. Strategies beyond producing coherent alignments need to be developed.

An interesting extension of the track may be to introduce different types of alignment relations in addition to equivalence. For instance, we may want systems to find subsumption relationships [146] well as part-of relationships which are important in anatomy ontologies. From an organizational point of view the main challenge will be to define the reference alignment. In addition to finding the correct correspondences, we also need to take into account how to deal with derivable correspondences in the computation of the evaluation measures. Regarding subsumption, traditional precision and recall may not be easily used, but we may need to use some variants of semantic precision and recall, e.g., [36, 52]. Regarding part-of, there are different kinds of partitive relations and the interaction with subsumption is not always straightforward [94].

Also when dealing with interactive matching, other evaluation measures may be used as in [127, 133]. Further, current evaluations focus on performance on the whole ontologies, but do not allow for comparing alignments of specific parts of ontologies, or for comparing alignments to the reference alignment at the detailed level of concepts and relations. Therefore, we could partition the ontologies into regions and evaluate on a more detailed level in order to gain a better understanding of the strengths and weaknesses of the systems^25^.

Although the OAEI requires a specific structure for the system papers in the proceedings of the Ontology Matching workshop, for some systems it was not easy to find details about the used strategies. Therefore, when possible, we may want to require a better description of the used strategies based on the different components in the ontology alignment systems.

## Endnotes

^1^ The proceedings for the Ontology Matching workshop since 2006 are available via http://ceur-ws.org/(volumes 225, 304, 431, 551, 689, 814, 946, 1111, 1317, 1545, 1766). The summary papers are titled ’Results of the Ontology Alignment Evaluation Initiative X’ with X the year of the workshop [1, 2, 7, 9, 34, 37–40, 54]. We made sure that the redundancy between this paper and the summary papers is minimal and when there is redundancy the data is used in a different way than in the summary papers.

^2^ We note that for the reader who only wants an overview of the lessons learned and most common observations that we have summarized these lessons in “Conclusion” - “Summary of observations” Section and also ***highlighted*** the relevant words and sentences in the other sections.

^3^ The precision of StringEquiv (2016) is 0.997, its recall 0.622 and its F-measure (with *α* = 1) 0.766.

^4^ An unsatisfiable concept is a concept to which no instance can belong. Its interpretation in model-based semantics is the empty set.

^5^ informatics.jax.org/expression.shtml

^6^ informatics.jax.org/searches/AMA_form.shtml

^7^ ncit.nci.nih.gov/ncitbrowser/

^8^ https://www.nlm.nih.gov/research/umls/

^9^ Provided by Martin Ringwald and Terry Hayamizu. See [64] for an overview of the mapping project including the development of the initial OAEI reference alignment as well as some further changes of which some are used in OAEI.

^10^ Thanks to Daniel Faria and Chris Mungall.

^11^ It was also included in 2011 but only one system implemented the necessary interface.

^12^ Although there are many issues related to user involvement [35, 72], currently only the influence of the validation of correspondences is taken into account in this track.

^13^ http://www.seals-project.eu/

^14^ A system that implements and evaluates several recommendation strategies can be found in [95].

^15^ We note that not all presented strategies are always used in the Anatomy track.

^16^ http://uberon.github.io/

^17^ http://bioportal.bioontology.org/

^18^ https://www.nlm.nih.gov/mesh/

^19^ http://sig.biostr.washington.edu/projects/fm/AboutFM.html

^20^ https://wordnet.princeton.edu/

^21^ On the OAEI web pages the second instance is called 2011.5, but in this paper we denote it as the ’2011 version’ of the track.

^22^ The bottom and top of the box (grey area) are the first and third quartiles. The band inside the box is the second quartile (the median). The ends of the whiskers represent the minimum and maximum of all of the data.

^23^ Although the task was not run anymore, TOAST presented results in their 2012 OAEI paper.

^24^ http://oaei.ontologymatching.org/2009/results/anatomy/

^25^ From 2017 the track organizers have the possibility to use an interactive system for visual exploration of ontology alignments [70].
